# Evaluating the clinical and cost-effectiveness of a conservative approach to oxygen therapy for invasively ventilated adults in intensive care: Protocol for the UK-ROX trial

**DOI:** 10.1177/17511437241239880

**Published:** 2024-04-11

**Authors:** Daniel S Martin, Tasnin Shahid, Doug W Gould, Alvin Richards-Belle, James C Doidge, Julie Camsooksai, Walton N Charles, Miriam Davey, Amelia Francis Johnson, Roger M Garrett, Michael PW Grocott, Joanne Jones, Lamprini Lampro, Lorna Miller, B Ronan O’Driscoll, Anthony J Rostron, Zia Sadique, Tamas Szakmany, Paul J Young, Kathryn M Rowan, David A Harrison, Paul R Mouncey

**Affiliations:** 1 Peninsula Medical School, University of Plymouth, Plymouth, UK; 2 Intensive Care Unit, University Hospitals Plymouth NHS Trust, Plymouth, UK; 3 Clinical Trials Unit, Intensive Care National Audit & Research Centre (ICNARC), Napier House, London, UK; 4 Critical Care, Maidstone and Tunbridge Wells NHS Foundation Trust, Kent, UK; 5 Patient and Public Representative, Bristol, UK; 6 Perioperative and Critical Care Theme, NIHR Southampton Biomedical Research Centre, University Hospital Southampton/University of Southampton, Southampton, UK; 7 Respiratory Medicine, Northern Care Alliance NHS Foundation Trust, Salford Royal University Hospital, Salford, UK; 8 Integrated Critical Care Unit, South Tyneside and Sunderland NHS Foundation Trust, Sunderland, UK; 9 Department of Health Services Research and Policy, London School of Hygiene & Tropical Medicine, London, UK; 10 Critical Care, Aneurin Bevan University Health Board, Cwmbran, UK; 11 Intensive Care Unit, Wellington Hospital, Wellington, New Zealand; 12 Medical Research Institute of New Zealand, Wellington, New Zealand; 13 Australian and New Zealand Intensive Care Research Centre, Monash University, Melbourne, VIC, Australia; 14 Department of Critical Care, University of Melbourne, Melbourne, VIC, Australia

**Keywords:** Oxygen, hypoxaemia, mechanical ventilation, intensive care, critical care

## Abstract

**Background:**

In the United Kingdom, around 184,000 adults are admitted to an intensive care unit (ICU) each year with over 30% receiving mechanical ventilation. Oxygen is the commonest therapeutic intervention provided to these patients but it is unclear how much oxygen should be administered for the best clinical outcomes.

**Methods:**

The UK-ROX trial will evaluate the clinical and cost-effectiveness of conservative oxygen therapy (the minimum oxygen concentration required to maintain an oxygen saturation of 90% ± 2%) versus usual oxygen therapy in critically ill adults receiving supplemental oxygen when invasively mechanically ventilated in ICUs in England, Wales and Northern Ireland. The trial will recruit 16,500 patients from approximately 100 UK adult ICUs. Using a deferred consent model, enrolled participants will be randomly allocated (1:1) to conservative or usual oxygen therapy until ICU discharge or 90 days after randomisation.

**Objectives:**

The primary clinical outcome is all cause mortality at 90 days following randomisation.

**Discussion:**

The UK-ROX trial has received ethical approval from the South Central – Oxford C Research Ethics Committee (Reference: 20/SC/0423) and the Confidentiality Advisory Group (Reference: 22/CAG/0154). The trial commenced in May 2021 and, at the time of publication, 95 sites had opened to recruitment.

## Background

Oxygen is the commonest therapeutic intervention administered to critically ill patients receiving mechanical ventilation, yet we do not know how best to titrate it in order to ensure optimal clinical outcomes. Traditionally, the desire to avoid hypoxaemia led to a relatively liberal use of oxygen on intensive care units (ICUs) and in some patients this resulted in hyperoxaemia. As our understanding of oxygen physiology has improved, we have become more aware of the potential harm that can occur when excessive oxygen is used.^1^ In response to this, the intensive care community has begun to focus its attention on determining the right dose of oxygen to give to patients, particularly those receiving mechanical ventilation.

In order to try and understand whether more or less oxygen will lead to improved outcomes in critically ill patients a number of retrospective studies^2–5^ and prospective clinical trials^6–14^ have been conducted. Results have been inconsistent, so the question remains unanswered.^15–17^ The variation in published results may be due to differences in methodological design between trials, such as their definition of ‘liberal’ or ‘conservative’ oxygen therapy regimens, differences in inclusion and exclusion criteria, and variation in reported outcome measures. Many small to moderate sized trials have shown no difference in outcomes between intervention and comparison groups,^6–9,12–14^ which could be explained by inadequate separation of oxygen exposure between intervention groups.^17^ Failure to achieve protocolised oxygenation targets has the potential to reduce the true effect of an intended intervention. Heterogeneity of treatment effect may also be a contributing factor, whereby there are differential risk:benefit ratios across the broad spectrum of patients managed on ICUs, leading to an overall apparent nil effect when all patients are evaluated together in summary statistics.^18^ In addition, any clinical benefit of a conservative oxygen strategy is likely to be relatively small, meaning that a large trial would be required to detect it.

We therefore set out to conduct a large-scale, multi-centre, randomised controlled trial (RCT) to address this evidence gap.

## Aim

The UK-ROX trial aims to evaluate the clinical and cost-effectiveness of conservative oxygen therapy (a peripheral arterial oxygen haemoglobin saturation (SpO_2_) target of 90% ± 2%) for mechanically ventilated adults admitted to ICUs in the United Kingdom.

## Methodology

This protocol was written according to the guidance in the Standard Protocol Items: Recommendations for Interventional Trials (SPIRIT) 2013 Statement.^19^ We previously conducted a small RCT to assess the feasibility of enrolling adult patients receiving mechanical ventilation into a definitive RCT, which informed the design on this trial.^20^

### Study design

UK-ROX is a multi-centre, data-enabled, registry-embedded, RCT with an internal pilot phase and integrated economic evaluation. The trial is embedded within Intensive Care National Audit & Research Centre (ICNARC) case mix programme (CMP), the national clinical audit for adult critical care which covers 100% of adult, general ICUs in England, Wales and Northern Ireland. Linking trial data to the CMP and other routinely collected healthcare data, ensures an efficient and economical trial design that enables large-scale recruitment. The internal pilot phase to review progress on site and patient recruitment, and separation between the groups was carried out over the first 6 months of the recruitment period.

#### Setting

Approximately 100 adult NHS ICUs that contribute to the ICNARC CMP across England, Wales and Northern Ireland.

#### Population

We will enrol patients within 12 h of fulfilling the eligibility criteria below:

##### Inclusion criteria

- Aged ⩾18 years
- Receiving invasive mechanical ventilation in the ICU following an unplanned ICU admission (i.e. not admitted after an elective procedure) OR invasive mechanical ventilation started in the ICU (i.e. the patient was intubated in the ICU)
- Receiving supplemental oxygen (fractional inspired concentration of oxygen (FIO_2_) >0.21) at the time of enrolment

##### Exclusion criteria

- Previously randomised into the UK-ROX trial in the last 90 days
- Currently receiving extracorporeal membrane oxygenation (ECMO)
- The treating clinician considers that one trial intervention arm is either indicated or contraindicated.

### Participant timeline

**Figure 1.**
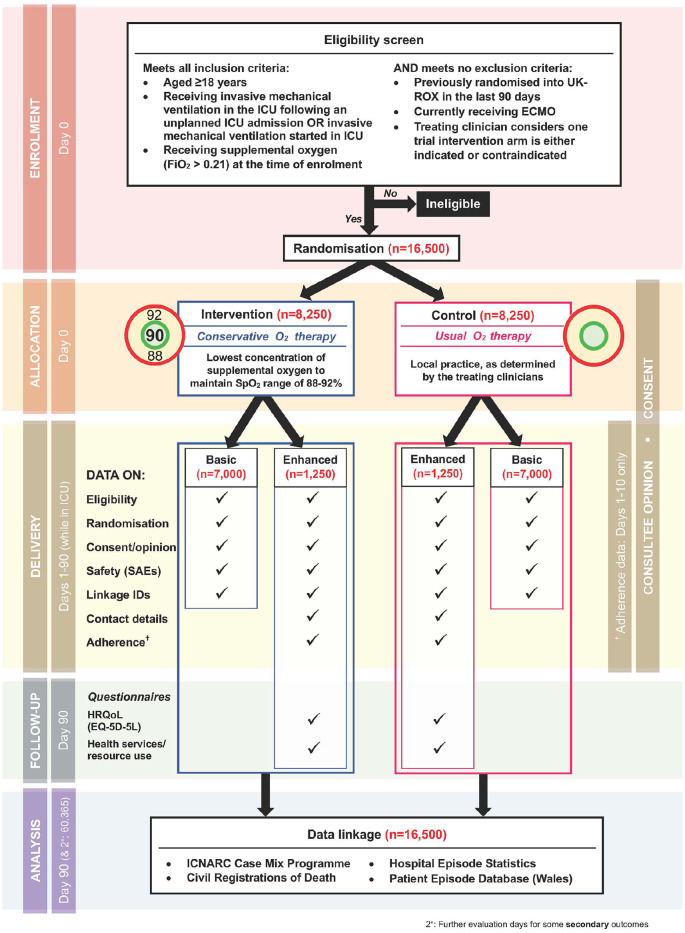
Trial flow diagram.

Figure 1 shows a flow diagram for the trial.

### Randomisation

Randomisation will occur as soon as possible after confirming participant eligibility. Participants will be randomised 1:1 to either intervention (conservative oxygen therapy) or comparator (usual oxygen therapy), using a central telephone or web-based randomisation service (https://www.sealedenvelope.com). Allocation will use randomised permuted blocks of variable block sizes, stratified by site, hypoxic ischaemic encephalopathy, sepsis and acute brain pathologies (excluding hypoxic ischaemic encephalopathy).

### Trial treatments

#### Intervention – Conservative oxygen therapy

**Figure 2.**
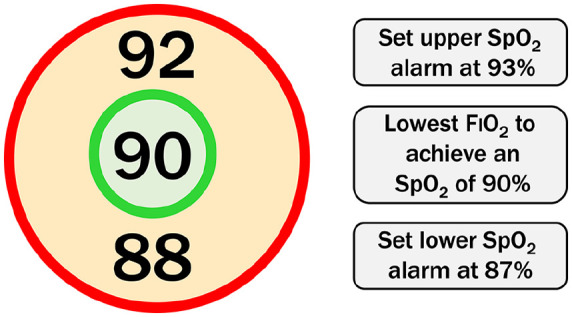
Conservative oxygen therapy intervention for patients receiving supplementary oxygen. Clinical team advised to maintain SpO_2_ at 90(±2)% that is, 88%–92% using the lowest FIO_2_ possible. The higher SpO_2_ limit was removed once patients were breathing an FIO_2_ of 0.21 (or room air). SpO_2_: Peripheral haemoglobin oxygen saturation; FIO_2_: fractional inspired oxygen concentration.

Conservative oxygen therapy is defined as the administration of the lowest concentration of oxygen possible to maintain a patient’s SpO_2_ at 90% ± 2%. Clinical teams are advised to continuously monitor SpO_2_ and titrate oxygen to achieve an SpO_2_ of 90%, whilst aiming to ensure SpO_2_ does not fall below 88% or rise above 92%. They are requested to use an SpO_2_ alarm that sounds if the SpO_2_ rises above 92% (Figure 2). The duration of the intervention is 90 days or until the patient is discharged from ICU, whichever is reached first. The SpO_2_ target should remain in place following extubation or the formation of a tracheostomy. Once oxygen is titrated down to 21% (room air), it may not be possible to maintain the SpO_2_ target and in this scenario, the upper SpO_2_ alarm should be deactivated.

#### Comparator – Usual oxygen therapy

Usual oxygen therapy is determined by the local clinical team, without any predefined limits set by the trial. The only request is that the use of an upper SpO_2_ alarm is avoided.

All other aspects of patient care will be at the discretion of the treating clinicians. If a trial participant is discharged from ICU and then readmitted within 90 days of enrolment, they should return to their allocated oxygenation group.

#### Protocol adherence

Protocol adherence will be monitored for a subset of patients in the conservative oxygen therapy group (see: *Data collection*). If the patient is receiving oxygen, a failure to reduce FIO_2_ when the SpO_2_ is above 92% (the upper limit of the SpO_2_ target range) for at least three consecutive hours defines a potential protocol deviation. Potential protocol deviations identified from the trial data will trigger a query to the participating site who will have the opportunity to provide a justification. In some cases (e.g. SpO_2_ values may have been above range only transiently on the hour but within range between the hourly recordings in the trial data), the Trial Management Group may determine that the event did not constitute a protocol deviation.

#### Blinding

Whilst not impossible, blinding of bedside clinical staff, members of the clinical research team and participants to the allocated oxygenation therapy group would be extremely challenging. SpO_2_ is an important monitor of clinical status so to conceal it from clinical staff could lead to unusual clinical decision-making and safely issue. Therefore, an open-label design was adopted.

#### Consent

The UK-ROX trial uses a research without prior consent (RWPC) model (also referred to as ‘deferred consent’), whereby eligible patients will be randomised to receive the assigned treatment as soon as possible under an Emergency Waiver of Consent under the Mental Capacity Act (approved by South Central – Oxford C Research Ethics Committee (reference: 20/SC/0423)). Following randomisation, and once the patient’s medical situation is no longer an emergency, a personal consultee opinion is sought to establish the patients’ likely wishes and feelings regarding participating in the trial. If there is no personal consultee available, a nominated consultee may be approached. Upon the participant’s recovery, they will be approached directly for informed deferred consent. The patient’s decision will be final, and will supercede the consultee, where there is disagreement. RWPC is an accepted consent model in adult emergency and critical care research where participants lack mental capacity and minimises the distress and additional burden on families during a distressing time.^21^ In addition, the urgent nature of treatments delivered in ICU means that any delay to commencing treatment could be detrimental to the patient (and to the scientific validity of the trial).

If a patient declines informed deferred consent, or a consultee advises that they believe the patient would not choose to participate in the trial, and, if a patient or their consultee (personal or nominated) withdraws consent/opinion at any time during the trial, this decision will be respected and will be abided by. All data up to the point of this decision will be retained in the trial unless the patient or consultee requests otherwise. Anonymised data necessary for the primary outcome will also be collected to avoid any potential bias from post-randomisation refusal of consent.

#### Safety monitoring

Adverse event (AE) reporting will follow the Health Research Authority guidelines on safety reporting in studies which do not use Investigational Medicinal Products (non-CTIMPs) (https://www.hra.nhs.uk/approvals-amendments/managing-your-approval/safety-reporting/). Occurrences of the following, pre-specified, adverse events are recorded for all randomised patients from the time of randomisation until ICU discharge or 90 days (whichever comes first): sinus tachycardia; supraventricular tachycardia; atrial fibrillation; myocardial ischaemia/infarction; and mesenteric ischaemia.

An event assessed as ‘severe’ or ‘life-threatening’ will be considered a serious adverse event (SAE) in the UK-ROX trial. Considering that all eligible patients are critically ill and at increased risk of experiencing multiple AEs to the complexity and severity of their condition,^22^ unexpected adverse events will are only recorded if they meet the criteria for an SAE and are considered to have occurred as a consequence of conservative oxygen therapy or usual oxygen therapy (i.e. deemed to be ‘possibly’, ‘probably’, or ‘definitely’ related to the trial procedures).

#### Follow-up

All patients will be followed-up to 90 days post-randomisation for the primary clinical outcome. A subset of patients (see data collection) will also be actively followed-up with a postal questionnaire containing the EuroQol EQ-5D-5L and a health services use questionnaire.^23^ Non-responders will be followed-up by telephone to confirm receipt and/or offer alternative methods of completion (e.g. over the telephone, via email).

#### Outcomes

Primary clinical outcome: 90 days all-cause mortality.

Primary economic outcomes: Incremental costs, quality-adjusted life years (QALYs) and net monetary benefit at 90 days.

Secondary clinical outcomes:

- ICU and hospital mortality (censored at 90 days)
- Mortality at 60 days and 1 year
- Duration of ICU and acute hospital stay (censored at 90 days)
- HrQoL, assessed using the EuroQol EQ-5D-5L questionnaire, at 90 days

Secondary economic outcomes:

- Resource use and costs at 90 days
- Estimated lifetime incremental cost-effectiveness.

### Data collection

Data collected is embedded within the routine data collection for the CMP national clinical audit. Minimal trial-specific data collection is required to confirm consent status for all randomised patients. For a subset of 2500 patients some additional in-patient clinical data (SpO_2_, FIO_2_ and arterial partial pressure of oxygen (PaO_2_) measurements) will be collected for intervention/adherence monitoring (Table 1). This will include prospective data for the first 10 patients at each site (to identify early issues and inform the internal pilot), followed by retrospective collection from randomly sampled patients across sites and treatment groups (identified to sites after the initial treatment period). Health-related quality of life (HrQoL) will be measured on the same sample and will require collection of patient contact details.

**Table 1.** Basic and enhanced primary data collection schedule.

	Level of data collection	
	Basic	Enhanced
Patients	14,000/16,500	2500/16,500
Collected in-hospital		
Eligibility/randomisation data	✓	✓
Consent/opinion data	✓	✓
Patient contact details		✓
Intervention/adherence data		✓
Serious Adverse Event (SAE) data	✓	✓
Collected at follow-up		
HrQoL (EQ-5D-5L) at 90 days		✓
Health services/resource use at 90 days		✓

All patients recruited to the trial will be consented for data linkage with other routine data sources, to obtain date of death occurring after acute hospital discharge by data linkage with civil death registrations and hospital costs for subsequent hospitalisations, by data linkage to hospital episode statistics (HES) and patient episodes data for Wales (PEDW).

### Statistical plan

#### Sample size

Based on data from potentially eligible patients in the CMP (*N* = 96,028, April 2017 to March 2019) and the Risk II study dataset^24^ (*N* = 82,075, April 2014 to March 2016), 90 days all-cause mortality is anticipated to be 37%.^24^ To detect an absolute risk reduction of 2.5% (relative risk reduction 6.8%) in 90 days all-cause mortality from 37.0 to 34.5% with 90% power requires a total sample size of 15,444 patients. Allowing for 6% refusal of consent/withdrawal/loss to follow-up (based on figures from one of our recently completed trials of critically ill patients in the UK^25^), we aim to recruit a total of 16,500 patients.

#### Clinical effectiveness analysis

All analyses will be lodged in a statistical analysis plan, a priori, before unblinding of investigators to any trial outcomes. All analyses will follow the intention-to-treat principle. Baseline patient characteristics will be compared between the two groups to observe balance and the success of randomisation. These comparisons will not be subject to statistical testing. The delivery of the intervention will be described in detail. Results will be reported in accordance with the CONSORT statement.

Analysis of the primary outcome (90 days all-cause mortality) will be performed both adjusted only for site, hypoxic ischaemic encephalopathy, acute brain pathologies (excluding hypoxic ischaemic encephalopathy) and sepsis (as stratification variables) and also adjusted for additional baseline covariates. Effect estimates will be estimated using regression models incorporating site random effects, and the absolute risk reduction and relative risk reported. Adjustment for baseline covariates can increase the precision of the estimate of treatment effect, and therefore the power of the trial, adjusting for any chance imbalance between the treatment groups. The covariates for inclusion in the adjusted analysis will be selected a priori based on established relationship with outcome for critically ill patients, and not because of observed imbalance, significance in univariable analyses or by stepwise selection method.

Analyses of secondary outcomes will use similar regression models with the binomial/Poisson family for binary outcomes and normal family for continuous outcomes. Analyses of duration of ICU and acute hospital stay will be performed by Wilcoxon rank-sum tests, stratified by survival status. Survival will be presented as Kaplan-Meier plots and analysed by Cox proportional hazards models with shared frailty at the site level.

Subgroup analyses will test for an interaction between treatment group and subgroup (for a limited number of subgroups specified a priori) in the adjusted regression models for the primary outcome. Key subgroups will be: suspected hypoxic-ischaemic encephalopathy; acute brain injury (excluding hypoxic-ischaemic encephalopathy); and sepsis.

Two interim analyses will be carried out after the recruitment and 90 days follow-up of 4500 and 10,000 patients using a Peto-Haybittle stopping rule (*p* < 0.001) to recommend early termination due to either effectiveness or harm. Further interim analyses will be performed if requested by the DMEC.

#### Cost effectiveness analysis

Information on resource use associated with the interventions and health-related quality of life will be obtained from detailed in-patient clinical data collected on the 15% of trial participants selected for enhanced data collection. A cost-effectiveness analysis (CEA) will be undertaken to assess the relative cost-effectiveness of the use of conservative oxygen therapy versus usual oxygen therapy according to the intention-to-treat principle. The CEA will take a health and personal health services perspective and will measure patient resource use and HrQoL outcomes over 90 days post-randomisation.

Patient level resource use and outcome data collected as a part of the trial will be linked with the CMP and HES databases and patient follow-up questionnaire will be used to report cost-effectiveness at 90 days. Regression models to predict resource use associated with the interventions, and the use of primary and community health services for all patients in the trial, will be developed. Patient-level resource use data will be combined with appropriate unit costs from the NHS payment by results database and Personal Social Services Research Unit to calculate total costs per patient for up to 90 days post-randomisation.

HrQoL at 90 days will be assessed from the enhanced data collection patients using the EuroQol EQ-5D-5L questionnaire, which will be valued using an appropriate EQ-5D-5L value set. HrQoL for all patients will be predicted by following a similar approach outlined for the costs as above. HrQoL data will be combined with the survival data to report QALYs at 90 days. QALYs will be calculated by valuing each patient’s survival time by their HrQoL at 90 days according to the ‘area under the curve’ approach. For 90 days survivors, QALYs will be calculated using the EQ-5D scores at 90 days, assuming an EQ-5D score of zero at randomisation, and a linear interpolation between randomisation and 90 days. For decedents between randomisation and 90 days, we will assume zero QALYs. Net monetary benefits will be calculated by valuing QALY gains at £20,000 per QALY and subtracting incremental costs.

The CEA will follow the intention-to-treat principle and report the mean (95% confidence interval) incremental costs, QALYs and net monetary benefit at 90 days since randomisation. The CEA will use multilevel linear regression models adjusting for key baseline covariates at both patient and site level as per the clinical effectiveness analysis. The CEA will perform extensive sensitivity analysis to check the robustness of cost-effectiveness results at 90 days. The cost-effectiveness results at 90 days will be reported across all subgroups as included for the clinical effectiveness analysis.

Lifetime cost-effectiveness will be projected by summarising the relative effects of alternative strategies on long-term survival, and HrQoL as compared with that of the age/sex matched general population. The survival of the patients who survive the initial acute hospital episode and all readmissions to the same critical care unit up 90 days post-randomisation will be extrapolated over a lifetime horizon. The extrapolation will assess the duration and magnitude of excess mortality of ICU patients relative to those of the age/sex matched general population, and will predict survival and HrQoL of the trial population for the period of excess mortality. After the period of excess mortality, age/sex matched general population survival and HrQoL will be applied. The lifetime costs will be projected by applying morbidity costs estimated at 90 days over the period of excess mortality. Predicted survival and HrQoL will be combined to report lifetime QALYs, and to project lifetime incremental costs, incremental QALYs, and incremental net benefits for the alternative strategies of care.

### Ethics and oversight

#### Ethical approval

The trial has received ethical approval from the South Central – Oxford C Research Ethics Committee (Reference: 20/SC/0423), approval from the Health Research Authority (Integrated Research Application System (IRAS) number: 260536) and a favourable opinion from the Confidentiality Advisory Group (Reference: 22/CAG/0154). The trial will be conducted in accordance with the: terms of the favourable ethical opinion; the approved trial protocol; ICH-GCP guidelines^26^; the UK Data Protection Act; the Mental Capacity Act; and ICNARC Clinical Trials Unit (CTU) research policies and procedures. The Sponsor is the Intensive Care National Audit & Research Centre (ICNARC).

#### Trial management

The trial management group (TMG), is responsible for the overall management of the UK-ROX trial, and is led by the Chief Investigators. The TMG comprises methodological, clinical and patient and public involvement (PPI) co-investigators as well as members of the ICNARC CTU trial team who coordinate the trial. Independent oversight is provided by an independent data monitoring and ethics committee (DMEC) and a majority independent (75% independent membership) trial steering committee (TSC).

## Discussion

The UK-ROX trial was designed to address an evidence gap and guide clinicians in choosing the most appropriate SpO_2_ targets in mechanically ventilated adult patients admitted to ICU. This multi-centre, open, data-enabled randomised clinical trial with internal pilot phase and integrated economic evaluation is powered to detect a 2.5% absolute difference in 90-day mortality, so should be able to answer whether or not conservative oxygen therapy is a clinically effective intervention when compared to usual practice. Given the large number of patients requiring mechanical ventilation in an ICU in the UK, even a small improvement in survival will equate to a large number of lives saved per year. The trial’s data-enabled, registry embedded design allows for a highly cost-effective approach to delivering a very large-scale trial within the NHS. The framework of the UK-ROX trial has been used to support two sub-studies. First is the *Exploring pulse oXimeter Accuracy across sKin Tones* (EXAKT) study, designed to determine the effect of skin tone on the diagnostic accuracy of pulse oximeters (NIHR135577/NCT05481515). Second is the *Mechanistic evaluation of two approaches to oxygen therapy in critical care* (MecROX) study, in which oxidative stress, redox status and surfactant metabolism biomarkers will be compared between participants in the two intervention groups of UK- ROX (NIHR151287/ISRCTN6192983). The UK-ROX trial is registered on the NIHR Associate Principal Investigator (PI) Scheme and 44 Associate PIs have already completed the 6 month training scheme.
